# In-Hospital Hip Fractures in a Large Irish Teaching Hospital: Patient Risk Factors and Outcomes

**DOI:** 10.7759/cureus.48931

**Published:** 2023-11-16

**Authors:** Matthew Condon, Alex Tofan, Tom McCarthy, Niall Hogan, Prasad Ellanti

**Affiliations:** 1 Department of Orthopaedics, St. James' Hospital, Dublin, IRL

**Keywords:** risk factors, outcomes, mortality, in-patient, hip fracture

## Abstract

Introduction: In-hospital hip fractures follow falls during unrelated admissions. Little data in the Irish setting is available on this vulnerable subset of hip fracture patients. Our objective is to review the incidence of in-hospital hip fractures, identify risk factors, and evaluate outcomes.

Methods: This is a retrospective observational review. We collected patient data in St. James’ Hospital using the Hospital In-Patient Enquiry database and Electronic Patient Records for in-hospital hip fractures between 10/02/2017 and 22/04/2020. Comorbidity, survival, and discharge destination data were gathered.

Results: We identified 40 fractures, representing 11.5% of all hip fractures treated at our center during the study period. The patients were 60-95 years old. Median age was 77 years for males and 86 years for females. Most (72.5%) were identified as fall risks, and 52% were unwitnessed falls. Many had a history of falls (67.5%), dementia (52.5%), or both (42.5%). Delirium was common (42.5%), and 75% had at least one vascular/coagulation disorder. Mortality was 10.25% at 30 days, 23.1% at 90 days, and 51.4% at 12 months. Although 70% were admitted from home, only 10% were discharged back home. 30% were admitted to a nursing home, and 55% were discharged from a nursing home.

Conclusion: In-hospital hip fractures accounted for 11.5% of all hip fractures treated at our center, confirming the need for a well-defined hospital protocol. Patients often present with previous falls, dementia, and cardiovascular disease. Outcomes are poor, with 51.4% mortality at 12 months and significant morbidity reflected by a loss of independent living.

## Introduction

In-hospital hip fractures are those that occur subsequent to a fall while patients are admitted for the management of other medical conditions. There is a paucity of data in the Irish setting regarding this particularly vulnerable subset of hip fracture patients.

Hip fractures are associated with an increase in all-cause mortality both immediately after the index event and in the long term, with the CHANCES project demonstrating a three-to-five-fold increase in all-cause mortality in the 3-6 months following the fracture and statistically significant differences in mortality for at least eight years following the event. Pre-existing chronic illnesses are also positively associated with poorer outcomes [1].

The Irish National Hip Fracture Database does not report on inpatient hip fracture rates [2]. However, the United Kingdom's (UK) National Hip Fracture Database (NHFD) reports these as 3.7% in 2019, 3.3% in 2020, and 3.0% in 2021. The in-hospital hip fracture rates show an overall downtrend since 2012 (5.2% in 2012 to 3.9% in 2018) [3]. There is significant variability between hospitals, with reported rates between 0.0% and 12.5% in 2016 [4]. While European countries have their own hip fracture databases, they mostly do not report specifically on hip fractures that occur as inpatients. 

The 2020 UK National Audit of Inpatient Falls (NAIF) report showed that patients who had suffered hip fractures during their inpatient stay demonstrated lower compliance rates for 5 of 6 key performance indicators, including time to orthogeriatric assessment, time to surgery, post-operative mobilization, rates of delirium, and destination of discharge, when compared to patients whose fractures occurred in the outpatient setting [5].

Mortality at 30 days for in-hospital hip fracture (IHF) patients is double that of non-IHF (12.7% compared to 5.8% for non-IHF) [5]. Studies suggest the increased mortality rate is sustained, with one large single-center study finding higher mortality at 30 days (11.4% vs. 6.7%), 90 days (22.6% vs. 12.6%), and 1 year (35.7% vs. 21.5%) [6]. Another study of UK patients over eight years found 30-day and one-year mortality rates almost doubled in the hospital subgroup (26% vs. 47% at one year), in addition to increased rates of co-morbidities and lower cognitive function [7]. The UK National Falls Audit also found certain risk factors were more common in IHF patients, including delirium, orthostatic hypotension, incontinence, visual impairment, and walking aids needed [8].

In-hospital hip fractures need to be tackled as part of a multidisciplinary effort to reduce their occurrence and the substantial associated morbidity and mortality. Risk groups need to be identified to allow these interventions to be implemented. As there is little data in an Irish context, we aimed to review the incidence of in-hospital hip fractures, identify potential risk factors, and define outcomes such as mortality and morbidity in our institution.

## Materials and methods

Conception

A significant portion of the hip fractures treated in the orthopaedic department in our center were noted to have occurred in the inpatient wards. As inpatient falls leading to fractures are not reported in our Irish National Hip Fracture Database as a percentage of total hip fractures, we aimed to establish the inpatient hip fracture rate in our center to illustrate this burden. This will ideally be the first of a series of audits in all Irish trauma centers to ultimately be incorporated in the data analysis of the National Hip Fracture Database.

Literature review

A review of the relevant literature was conducted focusing on our closest geographical and demographical neighbors in the UK, where the UK National Hip Fracture Database [3] reports the inpatient hip fracture rate and the National Audit of Inpatient Falls [5] reports the key performance indicators completion compared to falls in the outpatient setting. Comorbidities and mortality analyses are reported in large UK studies. To our knowledge, there are no Irish studies reporting the inpatient hip fracture rate, comorbidities, or outcomes.

Study design

This is a retrospective observational review. This was chosen because basic data was available in the electronic databases, and no intervention was conducted.

Data collection

We collected patient data in St. James’ Hospital, a large tertiary teaching hospital in Dublin, using the Hospital In-Patient Enquiry database and Electronic Patient Records (EPR) for in-hospital hip fractures between 10/02/2017 and 22/04/2020. Fractures suffered as inpatients were identified with a separate code on the HIPE database, and they were confirmed as such on review of the EPR. The total number of hip fractures treated in the center during that time period was also recorded from the HIPE database. EPR was searched for comorbidities as recorded by clinicians during inpatient stays or outpatient visits. Pre-admission residence and destination on discharge were included in the analysis. Survival time (post-fall) was recorded from EPR, and phone calls were made as necessary when data was incomplete in the electronic records. Where survival time was unclear due to missing records, the concerned patients were excluded from the mortality analysis. For example, if survival for 90 days was confirmed but not for 12 months, the patient was included in the 90-day analysis but not the 12-month analysis.

Data analysis

Demographic, comorbidity, and outcome data for each patient were compiled into spreadsheets. In the relevant cases, date of death and date of fall were compared to subclassify patients into 30-day, 90-day, 12-month, and >12-month mortality groups. From these, we reported our results mainly as proportions of the cohort population. The program Jamovi was used for the calculation of chi-squared values in the analysis of male vs. female mortality within our cohort.

## Results

We identified 40 fractures, which represent 11.5% of all hip fractures (n=347) treated at our center during the study period. There were 20 females and 20 males. Ages ranged from 60 to 95 years at the time of the fracture (mean age 81.4). Males presented at a median age of 77 years, whereas females presented at a median age of 86 years. Most falls resulting in fractures occurred on geriatric/rehabilitation wards (n=21, 52.5%) and medical wards (n=7, 17.5%), while seven occurred on surgical wards, two in the emergency department, and seven were unrecorded.

Many had a history of falls (n=27, 67.5%) or dementia (n=21, 52.5%), five of whom had previously documented behavioural and psychological symptoms of dementia (BPSD). Delirium was a common feature amongst these patients (n=17, 42.5%). A history of multiple previous fractures was documented in 27.5% (n=11) and postural hypotension in 15% (n=6). Hypertension (n=16, 40%), atrial fibrillation (n=12, 30%), ischaemic heart disease (n=10, 25%) and diabetes mellitus (n=9, 22.5%) were common co-morbidities amongst these patients. A majority (n=30, 75%) of patients had at least one major cardiovascular comorbidity. Osteoporosis was explicitly documented in only 20% (n=8) of patients in this high-risk group.

Excluding those who have been lost to follow-up and whose outcomes are uncertain (one prior to 30 days, five prior to 12 months), the 30-day mortality post-fall was 10.25% (4/39), the 90-day mortality was 23.1% (9/39), and the 12-month mortality was 51.4% (18/35). Even by optimistic standards (i.e., all those lost to follow-up have survived), 30-day mortality is 10%, 90-day mortality is 22.5%, and 12-month mortality is 45%. In our study, the 12-month mortality was 44.4% in women and 58.8% in men. Mortality rates by 30 days, 90 days, and 12 months are shown in Table 1. Note that two females and three males were excluded from the mortality analysis due to being lost to follow-up. Chi-squared statistical testing concluded with a confidence interval of 95% that this difference was statistically significant at 30 days (p=0.04) but not at 90 days (p=0.07) or at 12 months (p=0.395).

**Table 1:** Overall and comparative cumulative mortality of men and women at 30 days, 90 days, and 12 months

Time	Overall	Men	Women	X^2^; P value
30 days	N = 4, 10.3%	N=0, 0%	N=4, 20%	X^2^=4.23; p=0.04
90 days	N = 9, 23.1%	N=2, 10.5%	N=7, 35%	X^2^=3.29; p=0.07
12 months	N = 18, 51.4%	N=10, 58.8%	N=8, 44.4%	X^2^=0.734; p=0.395

**Figure 1:**
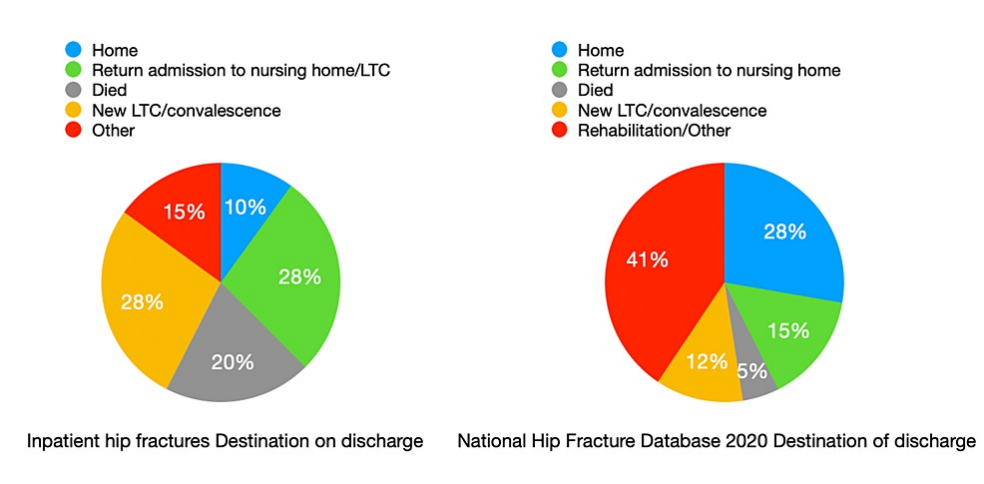
Destination of discharge of NHFD 2020 data vs. our inpatient hip fracture data

As an indirect measure of morbidity, 28 (70%) patients were admitted from home, whereas only four (10%) patients were discharged back home. Twelve (30%) patients were admitted from a nursing home; however, 22 (55%) patients were discharged to a nursing home or convalescent care. Figure 1 illustrates the differences in destination of discharge between our inpatient hip fracture cohort and the National Hip Fracture Database 2020 cohort of all hip fractures. Notably, the proportion of new long-term care (LTC)/convalescence and inpatient mortality is greater in our cohort than the NHFD 2020 data (28% vs. 12% and 20% vs. 5%, respectively), and the proportion of patients discharged home is lower in our cohort (10% vs. 28%).

## Discussion

In-hospital hip fractures accounted for 11.5% of all hip fractures treated at our center. As there is no Irish national data to contextualize this statistic, we look to the UK National Hip Fracture Database (NHFD), which reports an overall inpatient hip fracture rate during the time of our study of 4.1% in 2017 to 3.3% in 2020. Our inpatient hip fracture rates are in line with some hospitals in the UK, with reports of up to 12.5% [4], but the majority of hospitals included in the UK NHFD report rates below 5%. This high rate of 11.5% may be attributed to variations in patient cohorts between hospitals, as suggested in the 2016 UK NHFD report [4], but this cannot be determined until all acute trauma hospitals in Ireland are audited similarly and results are included in the Irish National Hip Fracture Database (INHFD), or a National Audit of Inpatient Falls (NAIF) as exists in the UK. This indicates an urgent need to audit all of Ireland’s acute hospitals to compare our data to other hospitals in Ireland and national data to UK data.

The high incidence rates of inpatient hip fractures also indicate the need for a well-defined protocol and audit standards, as exists with the Irish hip fracture standards or UK NHFD key performance indicators for community hip fractures. Green et al. called for a protocol for the management of inpatient hip fractures [9]. It is worth acknowledging that our hospital may demonstrate a relatively high incidence of inpatient hip fractures due to the large age-related and rehabilitation units, which in other hospitals are usually off-site.

In our study, hip fractures occurred almost a decade earlier in men than in women. Although hip fractures occur on average 3 to 6 years later in the community in women, the reason for the magnitude of the age difference in our data is unclear [10]. A large Danish study of gender differences in the evolution of time to first hospital admission finds only a one-year difference between men and women, showing the gender differences of inpatient hip fractures are not accounted for by the gender differences in first hospital admission [11].

Almost all of our patients had multiple co-morbidities, the most common being a previous history of falls, dementia, or delirium. Cardiovascular/coagulation-related co-morbidities were present in 75% of our patients, with high rates of hypertension, atrial fibrillation, IHD, T2DM, stroke, DVT, and peripheral ulcers. These co-morbidities not only indicate a high risk of falls (dementia, delirium, previous history of falls, atrial fibrillation/stroke, postural hypotension, OA) and a high risk of fracture (osteoporosis, previous history of multiple fractures), but also possible delays to surgery (HTN, IHD, diabetes, atrial fibrillation) and delayed recovery after surgery with medical co-morbidities delaying or complicating rehabilitation/discharge. The majority of fractures occurred in geriatric/rehabilitation or medical wards, likely due to the high prevalence of comorbid patients in these locations. High-risk locations should have targeted supports in place to minimize the burden of inpatient falls and hip fractures.

This patient cohort often has multiple co-morbidities and is well documented in the literature, with dementia/reduced Mini Mental State Exam (MMSE) scores [12-14], delirium [7,12-14], previous history of falls [7,13], higher ASA grade [15], T2DM [7], stroke [7,12,16], Parkinson’s disease [12], COPD [12], CKD [7], cancer [7], and polypharmacy [7]. These co-morbidities are significantly more common in the inpatient hip fracture group than in the community hip fracture group.

The risk represented by a previous history of falls is well mirrored in a large UK study, in which 21% (n = 68) of patients had been initially admitted to a hospital with falls/recurrent falls and then fell again. In this study, 59% (n = 192) had a documented previous history of falls, and 22% (n = 73) had suffered previous fragility fractures [7].

Outcomes are poor, with a 51.4% mortality rate at 12 months. The UK NHFD does not report on 12-month mortality; however, individual studies do. Large multi-year studies from the UK observe a one-year mortality rate ranging between 47% (vs. 26% in the community hip fracture control group) and 56.5% [7,17]. A 2010 meta-analysis found the overall unadjusted one-year mortality in all hip fracture patients to be 21.2% [18]. An analysis of recent national audits across the globe reports a mean one-year mortality rate for Europe of 23.3%. It is evident at one year that mortality among inpatient hip fracture patients is approximately double that of community hip fracture patients; this is also demonstrated in a comparative study [7].

At 30 days, the mortality in our study was 10.25%. Findings seem to vary significantly at 30 days between studies. Singh et al. found a 30-day mortality of 23.7% for those suffering inpatient hip fractures [17], whereas Johal et al. found a 30-day mortality of around 18%, double that of those suffering hip fractures in the community [7]. The UK NHFD reports the 30-day mortality quarterly national average as 6.1%-7.4% for the examined time period in patients suffering hip fractures in the community [19]. The UK NAIF 2020 reports, based on 2018 NHFD data, that the 30-day mortality for IHF patients is 12.7%, twice as high as the non-IHF (5.8%) patients [5].

Data from individual papers and national audits suggests mortality for inpatient hip fractures is approximately doubled at 30 days and sustained up to 12 months. Mortality data is commonly only reported for a maximum of 12 months. The impact of this significant increase in mortality depends heavily on the frequency of inpatient hip fractures. Our study found a significant difference in mortality between women and men at 30 days (20% vs. 0%, p = 0.04), at 90 days (35% vs. 10.5%, p = 0.7), and at 12 months (44.4% vs. 58.8%, p = 0.395). These differences are not statistically significant. While the observed mortality rate is higher in women at 30 days and 90 days, there is a reversal in the mortality rate at 12 months, where it is higher in men (Table 1). Schnell et al. found one-year mortality for men and women of 26.8% and 19.7% in the overall hip fracture population, respectively [18]. Kannegaard et al. found that cumulative mortality at 12 months among hip fracture patients compared to the general population was 37.1% in men and 26.4% in women [20]. While published data seems to be in line with our results in finding that mortality in men is higher at 12 months, there is little data, to our knowledge, of comparative mortality in men and women who have suffered inpatient hip fractures.

The percentage of new admissions to a nursing home in the IHFD was 4% after a community hip fracture. While 28% were discharged directly home, another 28% needed off-site rehabilitation [2]. Despite 70% of the included patients in this study being admitted from home, only 10% were discharged directly home. New admissions to a nursing home or convalescent care unit are striking, with 27.5% being discharged to a nursing home or convalescent care unit (Figure 1). These data show that patients who suffer hip fractures during their inpatient stay often have dramatically increased care needs, requiring transfer to a nursing home.

Note that our hospital has large on-site rehabilitation units and, therefore, would not target off-site rehabilitation. It would, therefore, not be relevant to compare rates of discharge to those of off-site rehabilitation units. The high inpatient mortality rate reflects the increased mortality post-inpatient hip fracture but may be higher than expected, given the capacity of our center for on-site rehabilitation. For the same reason, it is difficult to evaluate the increase in hospital length of stay. These variables specific to our center render it challenging to extrapolate our data to the rest of the country.

Limitations

Limitations of our study include missing data, with five out of 40 patients not having a recorded survival status at 12 months. This results in a 6.4% point margin of error for our mortality analysis at 12 months. Electronic patient records facilitate data collection but are subject to missing co-morbidities due to their reliance on clinicians recording these from clinical notes, particularly in the case of patients included in the study from 2017-2018 in the earlier stages of EPR. Comorbidities were not time-stamped, and some may not have been contributing to patients’ overall condition at the time of the fall, for example, in the case of delirium.

## Conclusions

Our study primarily demonstrates a high proportion of inpatient hip fractures in our center, with increased 30-day, 90-day, and 12-month mortality rates compared to community hip fractures. Our study also demonstrates at-risk patients are likely older, co-morbid patients with a history of falls, dementia, and other medical conditions and situated in medical or geriatric wards. Further research is needed if we are to ascertain any difference in mortality between men and women.

The UK NHFD and (more recently) the National Falls Audit report on inpatient hip fractures, and each hospital is audited to determine the inpatient hip fracture rates and follow-up mortality. The implementation of NAIF in the UK has demonstrated improvements in its key performance indicators. Our national audits currently do not include inpatient hip fractures despite the potentially high proportion of these fractures. As a result, we would recommend comprehensive audits be conducted in each orthopaedic center in Ireland to create a database similar to that in the UK. Our numbers demonstrate a clear need to audit other hospitals in Ireland. Inpatient hip fractures represent a significant functional burden, with high rates of new long-term care or convalescent admissions. A significant majority of the patients have either died or been admitted to a nursing home following an inpatient fall at the 12-month mark.
